# Impact of preoperative mild renal dysfunction on short term outcome in isolated Coronary Artery Bypass (CABG) patients

**DOI:** 10.4103/0972-5229.45075

**Published:** 2008

**Authors:** M. N. Ramakrishna, V. Deviprasad Hegde, G. N. Kumarswamy, Ratan Gupta, Narayana Swamy Moola, K. P. Suresh

**Affiliations:** **From:** Adult ITU, Narayana Hrudayalaya Institute of Medical Sciences, 258/A, Bommasandra Industrial Area, Anekal Taluk, Bangalore-560 099, India

**Keywords:** CABG-coronary arteries bypass graft, CPB-cardiopulmonary bypass, GFR-glomerular filtration rate, Mild renal dysfunction

## Abstract

**Background and Aim:**

It is well known that dialysis dependent renal failure increases the likelihood of poor outcome following cardiac surgery. But the results of CABG in patients with mild renal dysfunction are not clearly established. The aim of the study is to analyze the risk of preoperative mild renal dysfunction on outcome after isolated coronary surgery.

**Materials and Methods:**

We reviewed prospectively collected data between June 2006-Nov 2006 in 488 patients who underwent isolated CABG. We separated the data into two groups. Control group having normal renal function and study group having mild renal dysfunction (serum creatinine 1.4 mg-2.2 mg%). Among 488 patients, 412 patients were in control group and 76 patients were in the study group.

**Results:**

Analysis of data showed significant postoperative complications in the mild renal dysfunction group, like increased operative mortality (7.5% *vs* 1.6%), increased requirement of postoperative renal replacement therapy (10% *vs* 1.2%), increased incidence of new onset atrial fibrillation (20% *vs* 4.2%) and prolonged duration of ICU stay. Multivariate analysis adjusting for known risk factors confirmed preoperative mild renal dysfunction (S.creat.1.4-2.2 mg/dl) is an independent risk factor for postoperative morbidity and mortality. (Adj. OR: 4.47; 95% CI: 1.41-14.16; P=0.010).

**Conclusion:**

Mild renal dysfunction is an important independent predictor of outcome in terms of in-hospital mortality and morbidity in patients undergoing CABG.

## Introduction

Renal failure predisposes patients to adverse outcome after coronary artery bypass surgery. Moderate to end stage renal dysfunction is known to be an important predictor of morbidity and mortality after CABG and in this group a 5-year survival of less than 50% has been observed.[1] The two most common risk stratification scoring systems used to estimate perioperative mortality give a weighting factor only for advanced renal disease i.e. S.creatinine >200 umol/l or dialysis dependency.[2,3]

It is now recognized that even minor renal dysfunction as reflected by an increase in serum creatinine (or, more precise, by estimated GFR) on one hand and/or albuminuria or trace proteinuria on the other hand, has a major impact on cardiovascular risk.[4] Although a higher cardiovascular risk in patients with proteinuria had been recognized two decades ago,[5] both the consistency and the magnitude of cardiovascular risk which is associated with minor renal dysfunction have been fully appreciated only in the recent past.[6,7] Recently it is emphasized that increased risk of cardiovascular adverse events does exist even in patients with mild renal dysfunction and the magnitude of this risk is so large that there are recent efforts to give renal dysfunction the status of a major cardiac risk factor, similar to diabetes mellitus.[8,9] Several studies have shown that patients with mild renal dysfunction have an increased risk of mortality within 30 days of coronary surgery.[10,11]

There are several mechanisms[4] postulated through which impaired renal function causes cardiovascular morbidity such as:

- Early activation of sympathetic nervous system as a result of excitation of intrarenal chemoreceptors and mechanoreceptors that send activating signals into the hypothalamus where catecholamine turnover is increased leading to increased efferent sympathetic nerve traffic[12,13] (this may occur even when GFR is still normal).
- increased atherogenesis leading to oxidative stress[14]
- Early increase of blood pressure and left ventricular remodeling with evidence of impaired left ventricular diastolic function, insulin resistance, and a state of micro inflammation.

Increased concentration of an inhibitor nitric oxide synthetase-ADMA (asymmetric dimethyl-L- arginine).[15]

In the background of this information, it is imperative that special attention be focused on the outcome of CABG in patients with mild renal dysfunction. Few studies have been conducted in this context recently and have found significant risk of perioperative mortality among patients with mild renal dysfunction following CABG.[16,17] Results from these studies suggested that preoperative mild renal dysfunction is an independent predictor of mortality and morbidity in patients who undergo CABG.

Our study aims at investigating the impact of mild renal dysfunction (s.creatinine=1.4-2.2 mg/dl) in patients who have been undergoing elective isolated CABG in our hospital so that it provides some additional information regarding preoperative risk stratification.

## Materials and Methods

The study was conducted in patients undergoing isolated first time CABG at Narayana Hrudayalaya Institute of Medical Sciences. The data was collected prospectively as a part of preoperative risk stratification of patients undergoing CABG. Data were collected for a duration of six months between June 2006 to Nov 2006 and a total number of 488 patients were enrolled.

We analyzed data in all the patients undergoing first time isolated CABG with no history of renal disease or dialysis and with a preoperative serum creatinine >1.4 and < 2.2 mg/dl.

The patients were stratified into two groups:

1. Mild renal dysfunction group (S.creatinine 1.4-2.2mg/dl: N=76 )
2. Control group with normal renal function (S.creatinine < 1.4mg/dl: N=412)

The patients' glomerular filtration rate (GFR) was estimated using the Cockroft-Gault formula = (140 – age) × weight(kg)/(serum creatinine × 72 [×0.85 for women] and adjusted for each 1.73 m^2^ of body surface area. Renal dysfunction was defined as GFR< 60 mL/min per 1.73 m^2^ in accordance with the U.S. National Kidney Foundation guidelines.

### Statistical Analysis

Continuous data with normal distribution are given as mean ± standard deviation, otherwise as median, student t test for testing the significance of mean, *Chisquare* or Fisher exact test for testing the significance of percentages. OR –odds ratio and its 95% Confidence Interval have been used to find the relationship of outcome between two groups of patients. Multivariate analysis by logistic regression done for postoperative complications adjusting to various preoperative risk factors

If OR=1, outcome equally likely b/w two groups.

If OR>1, outcome is more likely in mild Renal dysfunction group

If OR< 1, outcome is less likely in mild Renal dysfunction group.

+ Suggestive significance 0.05<*P*< 0.10

* Moderately significant 0.01<*P* ≤ 0.05

** Strongly significant *P*≤0.01

Statistical software: The Statistical software namely SPSS 15.0, Medcalc 9.0.1, Stata 8.0 and Systat 8.0 were used for the analysis of the data and Microsoft word and Excel have been used to generate graphs, tables etc.

## Results

Preoperative patient characteristics are depicted in [Table 1].

**Table 1:** Comparison of study characteristics between normal RFT and mild renal dysfunction

Study characteristics	Normal RFT(n=412)	Mild Renal Dysfunction(n=76)	*P* value
Age	57.8 ±8.3	62.1±8.42	<0.001**
Sex	361:51	69:07	0.433
DM	189 (45.87%)	37 (48.68%)	0.652
HTN	167 (40.53%)	45 (59.21%)	0.003**
COPD	12 (2.91%)	3 (3.94%)	0.714
CVA	7 (1.69%)	4 (5.26%)	0.076+
PVD	4 (0.97%)	2 (2.63%)	0.237
SMOKER	94 (22.81%)	16 (21.06%)	0.735
RECENT MI	14 (3.39%)	4 (5.26%)	0.502
NYHA 4	36 (8.73%)	7 (9.21%)	0.894
EF >40%	370 (89.80%)	60 (78.94%)	0.007**
EF <40%	42 (10.19%)	16 (21.1%)	0.007**
PRE OP.ACE	71 (17.23%)	4 (5.26%)	0.008**
CABG			
ON CPB	120(29.12%)	20(26.3%)	
CPB(MINS)	100 ±41min	116 ±45	0.002**
ACX(MINS)	63.06 ±31.27	70.72 ±32.3	0.067+
BUN	11.34 ±3.47	16.65 ±6.81	<0.001**
S.CREATININE	1.02 ±0.16	1.61 ±0.2	<0.001**
GFR	78.44±16.26	51.30±9.53	

A total of 488 patients were analyzed of which 412 (84.4%) had serum creatinine less then 1.4 mg/dl and 76 (15.6%) were in the range of 1.4-2.2 mg/dl. Mean serum creatinine among the normal renal function group was 1.02±0.16 mg/dl and with mild renal dysfunction group was 1.61±0.2 mg/dl. The calculated glomerular filtration rate in renal dysfunction group was 51.30±9.53 ml/min/1.73 m^2^ and 78.44±16.26 ml/min/1.73 m^2^. 29.12% of patients underwent surgery on CPB which was comparable to mild renal dysfunction group i.e. 26.3%.

Analysis of the final postoperative outcome [Table 2] revealed significant complications among the mild renal dysfunction group compared to the normal renal function group. Postoperative mortality was higher among renal group compared to normal group (7.5% vs.1.65%, OR=4.95), and the requirement of postoperative RRT was significantly higher in mild renal dysfunction group (renal -10%, normal-1.2%, OR=9.58). Another striking finding was the incidence of postoperative atrial fibrillation which was significantly high & the incidence was more than three times in the mild renal dysfunction group compared to the normal renal function group (20% vs. 4.2%, OR=3.5) .Mean ICU stay was found to be significantly more in the renal group (4.76 days) as against the normal group (2.4 days). The need for reexplorations for various causes, the incidence of sepsis and the duration of mechanical ventilation were also found to be higher among the renal group even though it was statistically of moderate significance.

**Table 2:** Comparison of post operative outcome between normal RFT and mild renal dysfunction

Post-operative outcomes	Normal RFT(n=412)	Mild Renal Dysfunction(n=76)	*P* value	OR (95%CI)
Death	7 (1.6%)	6 (7.5%)	0.008**	4.95
				(1.62-15.19)
Post operative RRT	5 (1.2%)	8 (10%)	<0.001**	9.58
				(3.04-30.14)
Post op.AF	17 (4.2%)	15 (20%)	<0.001**	5.71
				(2.71-12.03)
Reexploration	19 (4.61%)	9 (11.84%)	0.012*	3.50
				(1.59-7.69)
IABP use(IOP&POP)	14 (3.39%)	5 (6.57%)	0.196	2.00
				(0.69-5.73)
Sepsis	12 (2.91%)	6 (7.89%)	0.046*	2.85
				(1.03-7.86)
ICU stay	2.4±1.4	4.76±7.46	<0.001**	-
Mechanical Ventilation	16.75 hrs	28.33 hrs	-	-

Finally, multivariate logistic regression analysis of postoperative outcome [Table 3] after adjusting for various preoperative risk factors revealed that mild renal dysfunction had significant positive relationship (Adj. OR-4.47, p=0.010, 95% CI;1.41-14.16 ) on poor outcome compared to other preoperative risk factors analyzed. Smoking (Adj. OR-2.87, p=0.001, 95% CI; 1.49-5.48) and recent myocardial infarction (Adj. OR-2.97, p=0.123, 95% CI; 0.75-11.86) were the other two preoperative risk factors which had a reasonable positive relationship to the poor outcome in this study.

**Table 3:** Multivariate logistic regression analysis of outcome/ complications for various preoperative variables

Risk factors	Logit	SE	Wald	P value	Adj. OR	95%CI
Mild renal dysfunction	1.50	0.59	6.48	0.010**	4.47	1.41-14.16
Male	-0.45	0.43	1.10	0.294	0.64	0.27-1.48
Age in years	0.03	0.02	3.40	0.065+	1.03	0.99-1.07
DM	0.18	0.29	0.40	0.528	1.20	0.68-2.09
Hypertension	0.04	0.30	0.01	0.904	1.04	0.58-1.86
COPD	-0.62	1.07	0.34	0.561	0.54	0.07-4.36
CVA	-0.47	1.17	0.16	0.685	0.62	0.06-6.17
Smoker	1.05	0.33	10.13	0.001**	2.87	1.49-5.48
MI<3 weeks	1.09	0.71	2.38	0.123	2.97	0.75-11.86
LV<40%	-0.33	0.53	0.39	0.531	0.72	0.26-2.02

## Discussion

Renal failure after cardiac surgery has a significant influence on postoperative morbidity and mortality. There is a complex interplay of a number of factors explaining renal failure associated with cardiac surgery.[18] There are factors relating to occult renal ischemia caused largely by arteriosclerosis and exacerbated by perioperative reduction of cardiac output, hypotension and resultant hypo perfusion.[19]

Rosita zaker *et al*[16] studied 4403 patients undergoing first time isolated CABG with preoperative serum creatinine less than 200 umol/l and found significant in-hospital mortality (2.1% vs. 6.1%, p<0.001), new dialysis (0.8% *vs* 5.2%, p<0.001), arrhythmias (29% *vs* 39%) among the mild renal dysfunction group. They have also done multivariate analysis of data which proved mild renal dysfunction as an independent predictor of outcome after CABG.

Hitoshi hirose *et al*[11] studied 1725 patients undergoing CABG and concluded that mild renal dysfunction had prolonged postoperative recovery which is associated with more frequent occurrence of major complications (28.8% in renal group *vs* 10.7% in control group, p<0.001) and mortalities (6.8% in study group *vs* 0.5% in control group, *p*<0.0005).

Caterina Simon *et al*[18] analyzed the association between mild renal dysfunction and CABG. He concluded that in multivariate analysis, length of ITU stay (median 48h *vs* 24h), mechanical ventilation time (med. 8h *vs* 6h) were influenced by creatinine clearance proving mild renal dysfunction group as an independent predictor of ICU stay and mechanical ventilation time.

**Figure 1:**
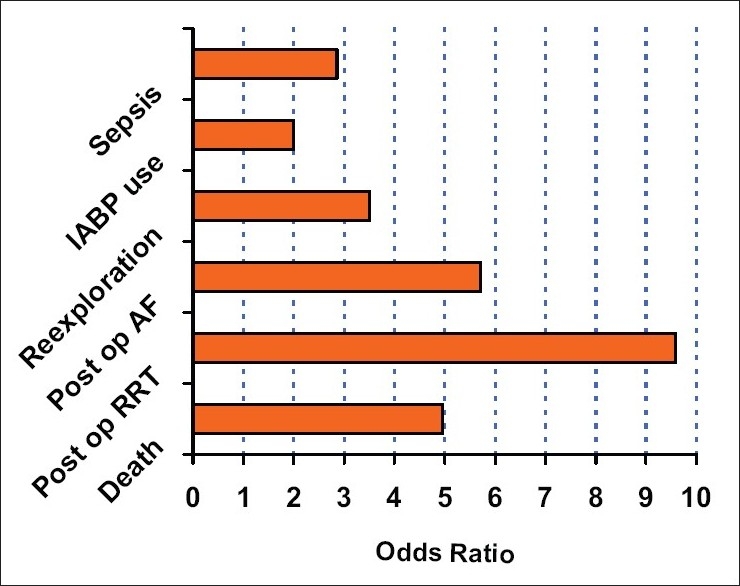
Risk of postoperative complications in renal group

In comparison with above studies, our study reveals significant post operative complications in the mild renal dysfunction group [Figure 1] especially ICU mortality (7.5% *vs* 1.6%), postoperative renal replacement therapy (10% *vs* 1.6%), atrial fibrillation (20% *vs* 4.2%), ICU stay (4.76 days *vs* 2.4 days). We have also observed increased rate of re-explorations, IABP use, sepsis and increased duration of mechanical ventilation in the mild renal dysfunction group even though these were statistically insignificant.

On further evaluation, the multivariate logistic regression analysis of complications using various risk factors supported our finding that mild renal dysfunction is indeed an independent preoperative risk factor for poor outcome after CABG.

## Conclusion

The findings of our study supports the hypothesis that mild renal dysfunction is an independent risk factor for an adverse outcome after CABG and carries significant mortality and morbidity. Hence the need for additional evaluation of these patients in order to understand the pathophysiology of the renal impairment and the design of renal protection strategies is required. Based on these data, we recommend that mild renal dysfunction (serum creatinine >1.5 or GFR in the form of creatinine clearance) be used as part of the process of preoperative risk stratification. Further work is required to develop models to integrate such data into existing or novel risk prediction tools.
